# DNA methylation dysregulation patterns in the 1p36 region instability

**DOI:** 10.1007/s13353-024-00913-9

**Published:** 2024-10-26

**Authors:** Joanna Swierkowska-Janc, Michal Kabza, Malgorzata Rydzanicz, Maciej Giefing, Rafal Ploski, Lisa G. Shaffer, Marzena Gajecka

**Affiliations:** 1 Institute of Human Genetics, Polish Academy of Sciences, Strzeszynska 32, 60-479 Poznan, Poland; 2 Chair and Department of Genetics and Pharmaceutical Microbiology, Poznan University of Medical Sciences, Poznan, Poland; 3 Department of Medical Genetics, Medical University of Warsaw, Warsaw, Poland; 4 Center for Reproductive Biology, College of Veterinary Medicine, Washington State University, Pullman, WA USA

**Keywords:** Monosomy 1p36 deletion syndrome, DNA methylation, Breakpoint 1p36 hotspot region, Chromosomal rearrangements, Chromosomal breakage, Genome instability

## Abstract

In the monosomy 1p36 deletion syndrome, the role of DNA methylation in the genomic stability of the 1p36 region remains elusive. We hypothesize that changes in the methylation pattern at the 1p36 breakpoint hotspot region influenced the chromosomal breakage leading to terminal deletions. From the monosomy 1p36 material collection, four cases with 4.0 to 5.5 Mb terminal deletions and their parents were investigated. DNA samples were assessed by targeted bisulfite sequencing (NimbleGen SeqCap Epi) to examine DNA methylation status in the 1p36 hotspot region at single-base resolution as compared to the chromosomal hotspot regions, 9p22, 18q21.1, and 22q11.2. Additionally, in in silico assessment, the mean GC content of various classes of repeats in the genome and especially in the breakpoint regions was evaluated. A complex landscape of DNA methylation in the 1p36 breakpoint hotspot region was found. Changes in DNA methylation level in the vicinity of the breakpoint in the child’s DNA when compared to parents’ and control DNA were observed, with a shift from 15.1 to 70.8% spanning the breakpoint region. In the main classes of evaluated repeats, higher mean GC contents in the 1p36 breakpoint region (47.06%), 22q11.2 (48.47%), and 18q21.1 (44.21%) were found, compared to the rest of the genome (40.78%). The 9p22 region showed a lower GC content (39.42%) compared to the rest of the genome. Both dysregulation of DNA methylation and high GC content were found to be specific for the 1p36 breakpoint hotspot region suggesting that methylation abnormalities could contribute to aberrations at 1p36.

**Supplementary Information:**

The online version contains supplementary material available at 10.1007/s13353-024-00913-9.

## Introduction

In the monosomy 1p36 deletion syndrome, chromosomal breakpoints are identified in telomeric or subtelomeric regions, resulting in various types of chromosomal rearrangements at 1p36 including terminal or interstitial deletions, translocations, and complex aberrations (Ballif et al. 2004b; Gajecka et al. 2006c, 2007, 2008). In our previous investigations of constitutional 1p36 rearrangements, a substantial number of the breakpoints were identified 4.0 to 5.5 Mb from the 1p36 telomere (Gajecka et al. 2007, 2008). In this hotspot, the breakpoints were frequently recognized in both maternally and paternally derived 1p36 deletions (Gajecka et al. 2007).

To date, several mechanisms of chromosomal rearrangements at 1p36 have been discussed (Ballif et al. 2004a; Gajecka et al. 2006a, 2008; D’Angelo et al. 2009; Zanardo et al. 2014). Despite the extensive effort to characterize the breakpoints at the molecular level and to identify sequence features and motifs involved in the 1p36 rearrangement formation, the general causative mechanism remains to be elucidated.

The link between abnormal DNA methylation patterns and genomic rearrangements other than those involving the 1p36 region has been identified in numerous studies, due to the impossibility of testing in germ cells, carried out in somatic cells (Carbone et al. 2009; Tang et al. 2012; Brabson et al. 2021). Although the role of DNA methylation in the genomic stability of the 1p36 region in cancer has been studied (Mori et al. 2017; Titus et al. 2017), there is no information available for constitutional monosomy 1p36. Therefore, we selected monosomy 1p36 cases with terminal deletions from our monosomy 1p36 material collection (Gajecka et al. 2007), and the DNA samples were assessed by targeted bisulfite sequencing (tBS–Seq) to examine methylation status in the 1p36 rearrangement hotspot region based on the patients and their parents (trio model). We hypothesize that dysregulation of DNA methylation pattern in gametes or early embryogenesis could predispose or lead to the chromosomal breakage at 1p36, resulting in constitutional rearrangements. At the same time, the mean GC content of various classes of repeats (long terminal repeats (LTR), long or short interspersed nuclear elements (LINE, SINE), simple repeats and low complexity regions) at the individual breakpoint regions as compared to the whole genome, including the other frequently rearranged chromosomal regions (9p22, 18q21.1, and 22q11.2) (Bogdanowicz et al. 2010; Simioni et al. 2010; Koczkowska et al. 2017), were evaluated in silico in the 1p36 constitutional chromosomal abnormalities to further assess the role of the DNA structural features in the chromosomal rearrangement formation process.

## Material and methods

### Material and the 1p36 rearrangement hotspot

Numerous breakpoints in all four classes of 1p36 deletions, localized at 4.0 to 5.5 Mb from the 1p36 telomere, were observed in our previously ascertained group of 145 patients with monosomy 1p36 (Supplementary Table S1).

To assure the homogeneity of the study group, only cases with terminal deletions were chosen for further assessment. Four trios of female patients with 1p36 de novo deletions and their parents as well as control DNA samples were chosen for further study. All provided informed consent after the possible consequences of the study were explained, in accordance with the Declaration of Helsinki.

The breakpoint location at 1p36 in each patient was carefully assessed as previously described (Gajecka et al. 2006b). Briefly, comparative genomic hybridization microarray analysis was performed (Signature Genomic Laboratories, LLC, Spokane, WA, USA) and the results of the identified chromosomal abnormalities were confirmed by fluorescence in-situ hybridization (FISH) with probes corresponding to the breakpoints on 1p36 as previously described (Gajecka et al. 2006b). In each study subject, to identify the 1p36 breakpoint, numerous sequence-tagged site (STS) markers were designed and STS marker walking analyses were performed using hybrid DNA containing the chromosome 1 with the 1p36 terminal deletion. Hybrids were constructed as previously described (Gajecka et al. 2006b). Somatic cell hybrids containing either the deleted chromosome 1 or the normal chromosome 1 were obtained, and those with the deleted chromosome were identified by the screening with microsatellite markers in the STS marker walking analysis and FISH (Page and Shaffer 1997; Gajecka et al. 2006b). Parental DNA samples derived from peripheral blood, DNA extracted from hybrids containing the normal chromosome 1 for each case, and hamster DNA were simultaneously tested in the STS marker walking to assure specificity of applied primers.

### Genomic sequences and annotations

The human genome sequence (GRCh37/hg19) was downloaded from UCSC Genome Browser (https://genome.ucsc.edu/). Additional annotations were obtained from UCSC (repeat element annotations, CpG islands) and Ensembl (gene and transcript annotations) (https://www.ensembl.org/index.html) databases.

### Analysis of sequence GC content

The GC content of 1p36, and other frequently rearranged chromosomal regions, 9p22, 18q21.1, and 22q11.2, were compared to the whole genome GC content distribution obtained by in silico scanning human genome sequence with non-overlapping 1 Mb windows. Windows that showed high fraction (> 10%) of unidentified bases (Ns) were discarded from the analysis. Repeat annotations were downloaded from the UCSC Genome Browser (GRCh37/hg19 human genome assembly, RepeatMasker track) and divided into main repeat classes (LTR, LINE, SINE, DNA, simple repeats and low complexity regions). The Wilcoxon rank sum test was used to compare the mean GC content of every class of repeats in the whole genome and in individual breakpoint regions. All statistical computations were performed using R programming language.

### Detection of low–copy repeats (segmental duplications)

The criteria described in the paper by Dittwald et al. (2013) were used to search for inverse paralogous low-copy repeats (IP–LCRs) in the whole 1p36.33-p36.11 region (chr1:1–28,000,000 bp). 1p36 region sequence was aligned to itself using BLAST (Camacho et al. 2009) and IP–LCRs were detected as non–overlapping regions of sequence similarity that showed following traits: the length of at least 1 kb, over 95% sequence identity and being located on opposite strands with the distance less than 10 Mb. Similar approach was utilized to detect “distant” IP–LCRs (no restrictions regarding similarity region distance) and direct low–copy repeats (LCRs) (located on the same strand). Separate similarity searches were performed between 1p36 and the ends of chromosomes 9, 12, and 22 (12 Mb of telomeric sequences of each chromosome) to detect terminal LCRs. As before, the requirements included the length of at least 1 kb and 95% sequence identity.

### Targeted bisulfite sequencing of the 1p36 region

Targeted bisulfite sequencing was applied to analyze DNA methylation of 1p36 breakpoint hotspot regions at a single-base resolution in patients with monosomy 1p36 and their parents. DNA probes extracted from peripheral blood samples derived from three trios (1p36_21, 1p36_41, and 1p36_56) and one duo (1p36_62) were assessed in the methylation assays. The NimbleGen SeqCap Epi Enrichment System (Wendt et al. 2018) for tBS–Seq was used. The probes were designed to cover the chr1:2,000,000–7,000,000 region (the primary target region), then during designing the probes, special attention was given to regions of the previously identified 1p36 breakpoints (Supplementary Table S1). The final regions covered by at least one probe are listed in Supplementary Table S2.

#### Library preparation and sequencing

For each sample, 1 µg of genomic DNA was fragmented on Covaris M220 (Covaris, Woburn, MA, USA) to an average size range of 180–220 bp. Libraries were prepared using the KAPA Library Preparation Kit Illumina (Kapa Biosystems, Wilmington, MA, USA) and SeqCap Epi Choice Enrichment Kit (Roche NimbleGen, Madison, WI, USA). Bisulfite conversion was done using EZ DNA Methylation-Lightning™ Kit (Zymo Research, Irvine, CA, USA) according to the manufacturer’s instructions. Libraries were paired-end sequenced (2 × 100 bp) on HiSeq1500 (Illumina, San Diego, CA, USA).

#### Bioinformatics analysis of 1p36 region DNA methylation

Adapter trimming and quality filtering of short reads obtained from bisulfite sequencing were conducted using Trimmomatic (Bolger et al. 2014). Only read pairs in which both reads had at least 50 bp after trimming were kept. Bismark (Krueger and Andrews 2011) was used to map filtered reads to the genome using directional alignment mode. Removing duplicated alignments from the mapped reads and cytosine methylation calling was performed using deduplicate_bismark and bismark_methylation_extractor programs from the Bismark package respectively. Methylation calling process was restricted to cytosines in the CpG context with the coverage of at least 30 × . Moreover, the first 5 bp of each mapped read were excluded from the methylation calling based on M–Bias plots. Final data processing reports created by Bismark are registered in Mendeley Data repository, listed in the “Data availability” section.

Bisulfite sequencing efficiency was estimated using custom Python scripts utilizing lambda spike-in control (accession no. NC_001416). CalculateHsMetrics program from the Picard toolset (http://broadinstitute.github.io/picard) was used to obtain metrics describing the effectiveness of targeted enrichment, such as mean coverage of target region and fold enrichment (Supplementary Table S3).

Principal component analysis (PCA) and hierarchical clustering (Pearson correlation metric, Ward's linkage criterion) of analyzed tBS–Seq samples were performed based on methylation percentages of cytosines in the 1p36 breakpoint hotspot region (chr1:4,000,000–5,500,000) using R environment. Differential methylation analysis between samples/groups of samples was conducted using methylKit package (Akalin et al. 2012). Significant differences in methylation of individual cytosines and windows of 100 bp length located in the 1p36 breakpoint hotspot region were searched, considering *q*-value < 0.01 and the methylation percentage change of at least 25%.

### Validation using bisulfite pyrosequencing

**Fig. 1:**
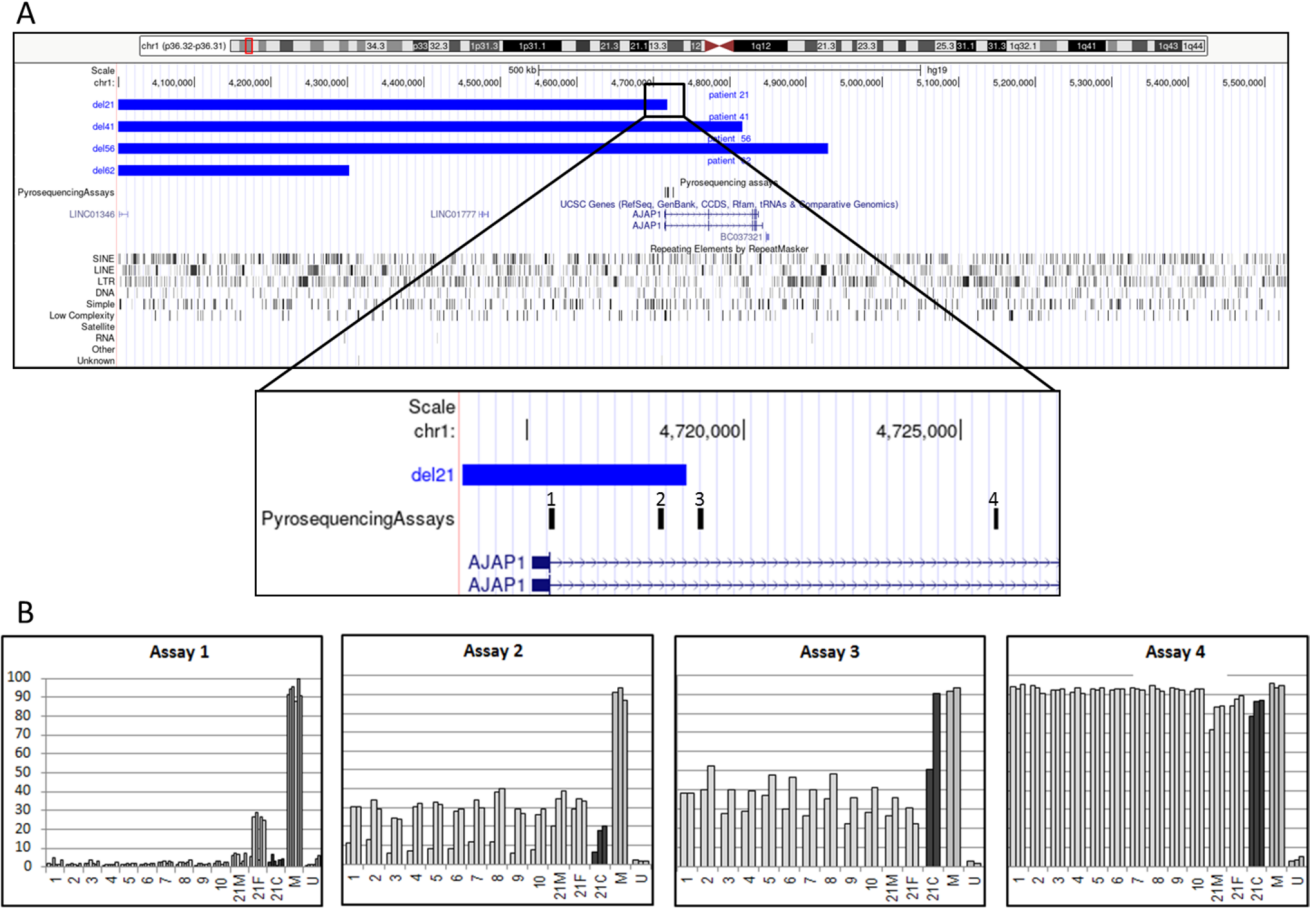
Deletions of monosomy 1p36 patients and designed bisulfite pyrosequencing assays for the child 21C from 1p36_21 family. **A** Amplicons for four specific assays (two of them were designed distally (1, 2) and two proximally (3, 4) to the breakpoint) of the 1p36.32 breakpoint *locus* for patient 21C from 1p36_21 family, assessed in the patient’s genomic DNA, are shown. **B** Methylation levels measured using the applied tests. Numbers 1–10 stand for control samples, 21M for a mother, 21F for a father, and a child (21C) is marked by dark grey color. M, methylated control; U, unmethylated control. The normal range of DNA methylation level for the assays is indicated in Supplementary Table S7

To validate the tBS–Seq results, DNA samples extracted from the 1p36_21 trio, and DNA samples derived from 10 unaffected individuals were evaluated using bisulfite pyrosequencing. Primers for four specific assays (two of them were designed distally and two proximally to the breakpoint) of the 1p36.32 breakpoint *locus* for patient 21C from 1p36_21 family were designed with PyroMark Assay Design Software 2.0 (Qiagen, Germany) (Fig. 1). The assays were verified for potential SNPs occurrence and CG sites within the primer binding regions. The primer and analyzed region details for all assays are summarized in Supplementary Table S4.

Purified DNA (500 ng) was converted with bisulfite solution according to EZ DNA Methylation Kit protocol (Zymo Research). PCR was performed according to PyroMark PCR kit (Qiagen) standard program: 95 °C for 15 min (initial heating), followed by 45 cycles: denaturation for 30 s in 94 °C, primer annealing temperature dependently on assay (Supplementary Table S4) for 30 s and extension in 72 °C for 30 s and the final extension in 72 °C for 10 min. PCR products were separated on 1.8% agarose gel stained with ethidium bromide and visualized under UV light (BioDoc-it Imaging System, UVR, USA).

Pyrosequencing was performed according to standard protocol and as described previously (Szaumkessel et al. 2011). In brief, PCR products were mixed with binding buffer (Qiagen) and sepharose coated with streptavidin (GE HealthCare, USA), shaken for 10 min and cleaned on vacuum pump station in the following buffers: 70% EtOH, 0.2% NaOH, and Washing buffer (Qiagen). Single strand amplicons were then mixed with annealing buffer (Qiagen) and the sequencing primer (0.4 µM), heated for 2 min at 85 °C, and cooled for primer hybridization. Pyrosequencing was performed using the Pyrosequencer PyroMark Q24 (Qiagen) and the results were analyzed using the PyroMark Q24 (2.0.6 Qiagen software), which automatically calculates the C/T (de facto mC/C*)* “ + ” strand or A/G on “ − ” strand (de facto C:mC) ratio at the CpG sites. Each pyrosequencing run was accompanied by fully methylated DNA control sample (CpG Genome Universal Millipore, Germany) and unmethylated Whole Genome Amplified (WGA) control sample prepared using the The GenomePlex® Whole Genome Amplification kit (Sigma-Aldrich, Germany) from DNA isolated from peripheral blood of 10 unaffected individuals. The results for each analyzed sample were visualized as value bars of DNA methylation level in each CG repeat separately.

To measure the wild-type range of DNA methylation level in the analyzed region, we used peripheral blood samples from 10 unaffected controls. We calculated the mean methylation values from assessed CG dinucleotides for each control sample. To indicate the cut-off level of hyper- and hypomethylation, the SD multiplied by two was added to the maximal methylation level from control samples (for hypermethylation) or subtracted from the minimal methylation level (for hypomethylation). Beyond these cut-offs, a sample was described as hypermethylated or hypomethylated, respectively.

## Results

### Characterization of the 1p36 hotspot of breakpoints

In 43 of 145 monosomy 1p36 cases, the rearrangement breakpoint(s) were localized in the hotspot. Of these 43, breakpoints involved in all types of chromosomal aberrations at 1p36 were observed: 23 terminal deletions, 5 interstitial deletions, and 15 translocations/complex aberrations (including derivative chromosomes). Eight breakpoints were previously experimentally narrowed at the DNA sequence level (Gajecka and Shaffer, 2008, data unpublished) in four paternally and four maternally derived terminal deletion cases as shown in Supplementary Table S1, from which four female cases were chosen for the methylation assessment.

### 1p36 region GC content

**Fig. 2:**
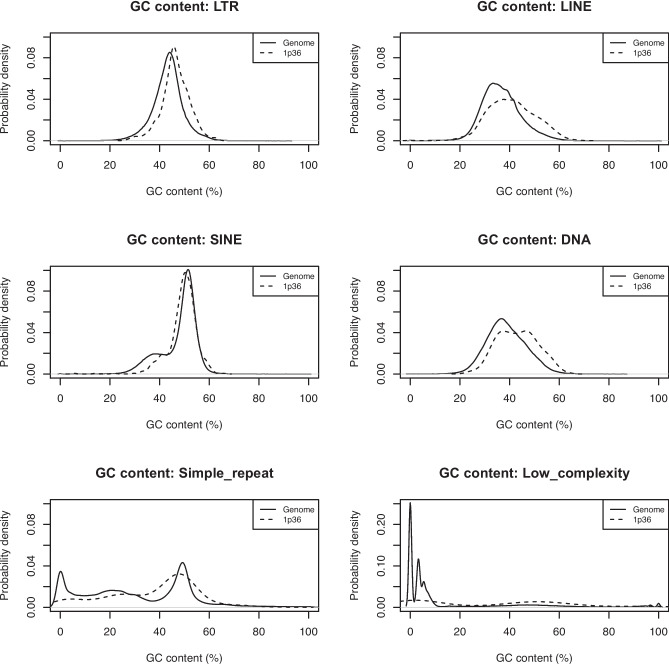
GC content distributions of different classes of repeat elements (LTR, LINE, SINE, DNA, simple repeats, and low complexity regions) in the whole genome and 1p36 breakpoint region (chr1:4,000,000–5,500,000)

We compared the mean sequence GC content of different classes of repeat elements in the whole genome and 1p36 breakpoint region (chr1:4,000,000–5,500,000) using the Wilcoxon rank sum test (Table 1). GC content distributions of main classes of repeat elements are presented in Fig. 2. We also performed the repeat element GC content analysis on other, frequent genomic rearrangement hotspots, 9p22, 18q21.1, and 22q11.2. While 22q11.2 and 18q21.1 hotspot regions showed similar GC content patterns to 1p36 region, 9p22 region exhibited significantly lower GC content compared to the genomic average (Supplementary Table S5).

**Table 1:** Comparison of sequence GC content of different classes of repeat elements in the whole genome and the 1p36 breakpoint region

Repeat class	GC content (genome)	GC content (1p36)	*p*-value
LTR	43.36%	46.41%	1.26713e − 49
LINE	36.74%	40.85%	1.82744e − 33
SINE	47.75%	49.26%	0.00595544
DNA	38.69%	43.13%	4.61849e − 13
Simple repeat	30.74%	37.25%	3.97042e − 08
Low complexity	15.12%	31.57%	3.73986e − 08

The analysis also indicated that genomic regions that show the highest GC content in the human genome (95th percentile and higher) tend to be located near chromosome ends—43.3% of them are placed within 5 Mb from an end of a chromosome and 58.9% are placed within 10 Mb.

### Low–copy repeats at 1p36.33-p36.11

The analysis of IP–LCRs showed that in the 1p36.33-p36.11 (chr1:1–28,000,000) IP–LCRs tend to be clustered in the following locations: chr1:100,000–700,000; chr1:12,800,000–13,700,000; chr1:16,800,000–17,200,000; chr1:21,700,000–21,800,000 and chr1:25,600,000–25,700,000, thus, outside the chr1:4,000,000–5,500,000 breakpoint hotspot region.

We also searched for other types of LCRs that might potentially be linked to non–allelic homologous recombination (NAHR) mediated genomic instability, including 'distant' IP–LCRs (no restrictions regarding the distance between the segments), direct LCRs (direct segmental duplications meeting the same length, similarity and distance criteria), as well as terminal LCRs (segments showing similarity to the ends of other chromosomes). In all cases, the analyzed LCRs were located in regions that significantly overlapped IP–LCR clusters.

### Differential DNA methylation at the 1p36 rearrangement hotspot region

**Fig. 3:**
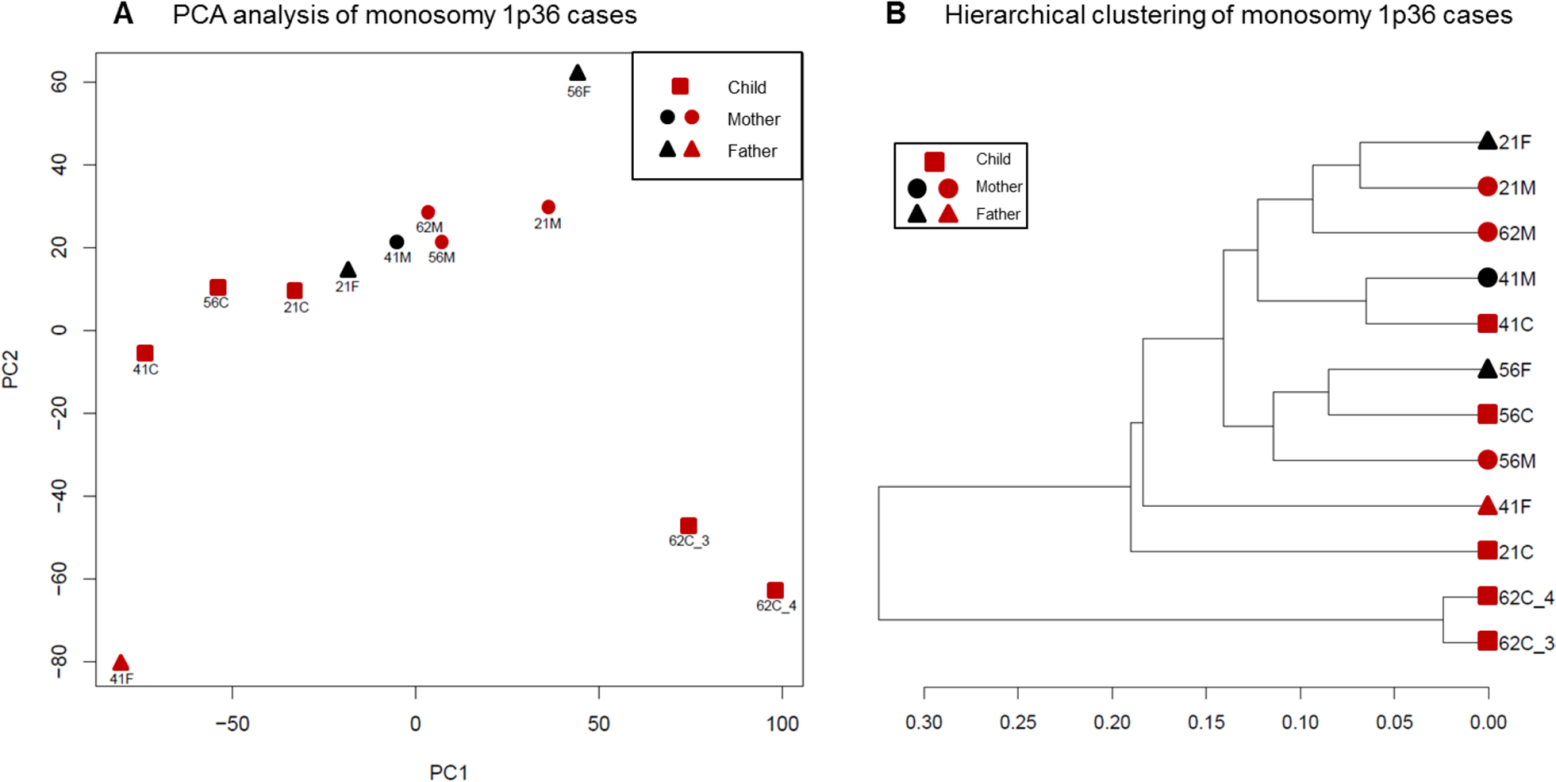
Results of the principal component analysis (PCA) (**A**) and hierarchical clustering (**B**) (Pearson correlation metric, Ward’s linkage criterion) of analyzed targeted bisulfite sequencing samples. Analyses were performed based on methylation percentages of cytosines in the 1p36 breakpoint hotspot region (chr1:4,000,000–5,500,000). Red color indicates the parent from whom the chromosome on which the deletion occurred de novo was inherited

Quality metrics of the tBS-Seq experiment are presented in Supplementary Table S3 and the raw data are available at the Mendeley Data repository. In order to examine if the assessed samples share the methylation patterns, we performed PCA and hierarchical clustering of samples based on methylation percentages of cytosines located in the chr1:4,000,000–5,500,000 region (Fig. 3). No simple relation was identified comparing the data obtained for the patients and their parents, with the analysis revealing a very complex relationship between 1p36 methylation in patients and their parents (Fig. 3). There seems to be no simple division between analyzed groups of samples visible in the PCA plot. The sample dendrogram shows that the 1p36 methylome of some patients, 41C and 56C, is highly similar to their parents, but not from whom the chromosome on which the deletion occurred de novo was inherited. Other patients, 21C and 62C, exhibit distinct DNA methylation patterns without similarities to their parents.

The results of the aforementioned exploratory analysis of the data clearly suggest that no meaningful results could be obtained from the group comparisons of the analyzed samples. Instead, we used methylKit (Akalin et al. 2012) to search for statistically significant differences at the single base and 100 bp window level, comparing individual patients and their parents. The numbers of identified differentially methylated cytosines and regions are presented in Supplementary Table S6. The results show that the highest number of differences at the single cytosine level can be generally found between patients and their parents from whom chromosomes with de novo breakpoints/deletion were inherited. Interestingly, different families seem to show different preferences for the type of methylation changes, with 1p36_41 and 1p36_56 families showing bias towards hypermethylation and 1p36_62 family showing bias towards hypomethylation. In case of 1p36_21 family, both types of changes, hypo- and hypermethylation, are present in roughly equal proportions. At the level of 100 bp regions, there are no substantial differences in DNA methylation level in numerical terms, but the prevalence of hyper- and hypomethylated sites has the same manner as at the single cytosine level. It can be at least partially explained by the fact that the identified differentially methylated cytosines are generally located outside regions of CpG concentration, with less than 3% of them overlapping known CpG islands.

### Methylation deregulation at the breakpoint hotspot region is confirmed by DNA bisulfite pyrosequencing

Results of the mean DNA methylation levels and cut-offs obtained in assays 1–4 are compiled in Supplementary Table S7. Four assays were designed to measure the mean methylation around and within the 1p36 deletions region in the family 1p36_21, in which the child inherited the chromosome on which the deletion occurred de novo from her mother. Assays designed for the studied region are marked in Fig. 1. Assays 1 and 2 correspond to chromosomal region deleted in the patient, while assays 3 and 4 correspond to the unchanged region localized proximally to the breakpoint. For the assay number 1, family 1p36_21 showed mean DNA methylation values: mother: 5.37%, father: 19.30% and child: 3.65%. For assay number 2, the family members showed a mean methylation of 31.26% in the mother, 32.40% in the father, and 15.11% in the child, which was relatively lower than in both parents and controls. In assays 1 and 2, only the region inherited from the father was analyzed, as the region inherited from the mother was deleted. The assay 3 resulted in 31.01% in the mother, 26.56% in the father, and 70.82% in the child, substantially higher than parental and control group. For the last assay (number 4) the family showed 79.92% methylation in the mother, 87.19% in the father, and 84.17% in the child. These values do not differ remarkably from the control group (Fig. 1).

## Discussion

The relationship between DNA methylation and genomic instability has been the subject of intensive investigation in the past few decades, especially in cancer research, but the connection between DNA methylation and genomic instability at the 1p36 region in constitutional chromosomal rearrangements remains to be elucidated. Both DNA hypomethylation and hypermethylation are associated with increased genomic instability that can lead to chromosomal rearrangements associated with malignant transformation (Brabson et al. 2021). DNA hypermethylation can lead to genomic instability by the inhibition of the recruitment of DNA repair proteins or a silencing of DNA repair genes (Toffolatti et al. 2014; Tsuboi et al. 2020; Brabson et al. 2021), and DNA hypomethylation can reduce the formation of heterochromatin leading to transposition of DNA at repetitive regions in the genome (Brabson et al. 2021). As repetitive sequences could be a source of genome instability, most of repetitive elements are usually methylated to maintain a heterochromatic state (Pappalardo and Barra 2021). It is known that alteration of the epigenetic pattern of repetitive sequences is characteristic of many complex diseases, thus it is difficult to understand if it is the cause or the consequence of the disease (Pappalardo and Barra 2021). However, there is increasing evidence showing that repetitive elements are frequently hypomethylated, which correlates with chromatin relaxation and unscheduled transcription, in various of human pathologies from cancer to psychiatric disorders (Lamprecht et al. 2010; Pappalardo and Barra 2021).

A possible explanation for the negative impact of hypomethylation of CpG–rich repeated elements on genomic stability has been proposed by De and Michor (De and Michor 2011). Significant enrichment of hypomethylated G-quadruplex sequences (G4s) in the near vicinity of somatic copy–number alteration breakpoints has been found, e.g., in many types of cancer, while information concerning this aspect in constitutional rearrangements involving 1p36 region has not been investigated. G-quadruplex structures are known to slow down the movement of DNA polymerase, increasing the likelihood of genomic rearrangements by such mechanisms as NAHR, non–homologous end joining (NHEJ), or fork stalling and template switching (FoSTeS) (Lupski 2015; Burssed et al. 2022). Their formation, however, depends on the open chromatin state and DNA accessibility, which is usually restricted by the hypermethylation of G4 sequences in normal tissues. Aberrant genome-wide DNA hypomethylation patterns resulting from carcinogenesis or a glitch in DNA methylation machinery suggested for the *Hylobatidae* family (Carbone et al. 2009) may therefore increase the occurrence of G4 structure formation and chromosome breaks in a tissue–specific manner (De and Michor 2011).

As far as we know, there are no published reports concerning abnormalities in methylation level in the monosomy 1p36 hotspot region. In the only article concerning methylation in a constitutional chromosomal syndrome, the haploinsufficiency of *SPEN* (NM_015001.3; chr1:16,174,202–16,266,951; GRCh37/hg19) was found to be associated with a distinctive X chromosome episignature in females with interstitial deletions at 1p36.21p36.13 (Radio et al. 2021). Although those reported 1p36 deletions do not overlap (12,700,001–20,400,000 bp) with our hotspot rearrangement region, patients have phenotypes similar to those seen in terminal 1p36 deletions such as intellectual disability, hypotonia, behavior abnormalities, multiple congenital anomalies, and facial dysmorphisms (Gajecka et al. 2007), and additionally obesity and increased BMI (Radio et al. 2021). Eleven individuals with interstitial deletions of 1p36.21p36.13 and truncating *SPEN* variants were included in the genome-wide methylation analyses, which indicated that *SPEN* haploinsufficiency was related to methylation changes on the X chromosome (Radio et al. 2021).

The human genome consists of different classes of repeats enriched with CpGs that are silenced into heterochromatic regions by both DNA methylation and repressive histone modifications (Brabson et al. 2021). The dynamic relationship between transcriptionally active subclasses of retroelements and DNA methylation has also been revealed, with abundant transcription of transposable elements serving as a boundary for changes in CpG methylation (Hoyt et al. 2022). The results of our analyses indicate that the 1p36 breakpoint hotspot region (chr1:4,000,000–5,500,000) meets all the prerequisite criteria for the aforementioned mechanism of a glitch in DNA methylation machinery, exhibiting high GC content and suggesting dysregulation of DNA methylation in monosomy 1p36 patients. Each class of repeat elements annotated in UCSC databases showed statistically significant higher GC content in the 1p36 breakpoint region compared to the rest of the genome, which is not surprising given its overall high GC content and high level of recombination reported for this chromosome region (D’Angelo et al. 2009). We found that the whole region has a mean GC content of 47.06%, which makes it at the 91st percentile in the human genome (40.78% mean GC content). A similar trend was observed in the 22q11.2 breakpoint region (48.47%, 94th percentile) and, to a lesser extent, the 18q21.1 region (44.21%, 80th percentile). Conversely, GC content of 9p22 region is significantly lower (39.42%, 44th percentile).

Our findings agree with the identified changes in methylation pattern in 22q11.2 deletion syndrome (Rooney et al. 2021). In genome-wide DNA methylation analyses of peripheral blood from 49 patients with 22q11.2 deletion syndrome, an evidence of a unique and highly specific episignature in typical and proximal 22q11.2 deletion syndromes was found (Rooney et al. 2021). The methylation pattern of patients with 22q11.2 deletion syndrome differed significantly from control individuals and > 1500 patients with other neurodevelopmental disorders with known episignatures (Rooney et al. 2021). Also, assessing the influence of DNA methylation on gene expression in the 22q11.2 hotspot, Starnawska et al. (2017) have suggested a relationship between changes in DNA methylation patterns at birth with development of a psychiatric disorder later in life in patients with 22q11.2 deletion syndrome. In that study, one CpG site, mapped to the *STK32C* gene, was associated with a later psychiatric diagnosis (Starnawska et al. 2017). In addition, differentially methylated CG dinucleotides in *LRP2BP*, *TOP1*, *NOSIP*, and *SEMA4B* were associated with intellectual disability, behavioral disorders, disorders of psychological development, and schizophrenia spectrum disorders, respectively (Starnawska et al. 2017). Pathway analysis of these genes indicated several pathways such as neurogenesis, neuron development, neuron projection development, astrocyte development, axonogenesis, and axon guidance as significantly enriched (Starnawska et al. 2017). In another study, it was found that methylation alterations in specific imprinting genes and in genes located in 6p21-p22, within the major histocompatibility complex (MHC) *locus*, might contribute to the development of schizophrenia spectrum disorders in 22q11.2 deletion syndrome (Carmel et al. 2021). Sixteen adult men with/without schizophrenia spectrum disorders were recruited from a 22q11.2 deletion syndrome cohort and underwent genome-wide DNA methylation analysis (Carmel et al. 2021). Differentially methylated probes and regions were enriched in two gene sets, “imprinting genes” and “chr6p21,” a region overlapping the MHC *locus* (Carmel et al. 2021). Most of the identified differentially methylated genes are involved in neurodevelopment and synaptic plasticity (Carmel et al. 2021).

Here, we found a complex landscape of DNA methylation at the 1p36 breakpoint hotspot region. While very few patterns can be observed in the data, the differences between patients and the parents from whom the de novo breakpoints were derived seem to be much higher than comparisons between the two parents. Moreover, these differences exhibit family–specific trends, showing either a preference for hyper- or hypomethylation changes, or both, or non-remarkable changes in DNA methylation.

It is important to remember that constitutional chromosomal and epigenetic rearrangements do not arise throughout a lifetime as in various cancers, but occur de novo in gametogenesis or early embryogenesis. It is unclear when the changes in methylation level could happen, either during germ cell development or during early embryogenesis, when global demethylation and remethylation take place. Epigenetic reprogramming in the germ cells involves the erasure of somatic methylation patterns in primordial germ cells and establishment of sex-specific germ cell methylation patterns (Zeng and Chen 2019). Whereas, reprogramming in early embryogenesis involves erasure of most methylation marks inherited from the gametes (Zeng and Chen 2019). A glitch in DNA methylation during one of those processes might predispose or lead to the chromosomal breakage. Beside embryo development and genome stability, DNA methylation is also involved in genomic imprinting, inactivation of X chromosome and repression of transposable elements (Bird 2002). In genomic imprinting only one of the two inherited alleles is expressed (monoallelic gene expression) and DNA methylation establishes imprinting marks on either paternal or maternal alleles (Elhamamsy 2017).

Another argument favoring a role of altered methylation in deletion formation is the lack of sequence features traditionally associated with genomic instability in the 1p36 breakpoint hotspot region. Previous analyses showed that motifs typically involved in genomic instability (translin sites, DNA polymerase a/b, frameshift hotspots, deletion hotspot, consensus sequences, etc.) are present in the vicinity of 1p36 breakpoint junctions but not significantly enriched (Gajecka et al. 2008). Two other important classes of genomic instability related features are direct LCRs and IP–LCRs that are known to be involved in genomic rearrangements via NAHR (Dittwald et al. 2013). We screened the whole 1p36 region (chr1:1–28,000,000) for their presence utilizing commonly used criteria (Dittwald et al. 2013). Overall, none of the LCR clusters found seem to be located in near vicinity of the 1p36 breakpoint hotspot region (chr1:4,000,000–5,500,000), which makes their participation in 1p36 genomic instability very unlikely. However, as reported by D’Angelo et al. (2009), in two terminal deletions of 1p36 that were about 25 kb apart, the breakpoint regions were refined to a genomic interval containing a series of 1p36 specific segmental duplications with 90–98% identity (D’Angelo et al. 2009), which may suggest a role in the rearrangements. Their results are consistent with previous findings indicating that genomic rearrangements directly mediated by LCRs via NAHR tend to be recurrent, sharing common size and breakpoint locations (Gu et al. 2008). Despite this, previously assessed, nonrecurrent 1p36 genomic breakpoints (Gu et al. 2008; Burssed et al. 2022), seem to be mediated by multiple mechanisms such as NHEJ (Gajecka et al. 2008) and FoSTeS (Lupski 2015).

The observed changes of DNA methylation levels in the vicinity of the breakpoint are intriguing. These results were found by the methylation array as well as pyrosequencing assays (assays 2 and 3). Assay 2 indicates a relatively lower level of methylation and assay 3 showed substantially higher level of methylation in the child as compared to the parents as well as control samples from healthy individuals. These results highlight a shift in methylation level from 15.11% (assay 2) to 70.82% (assay 3) spanning the breakpoint region. As loss of methylation results in higher genetic instability, a phenomenon frequently observed in cancer, it is tempting to speculate that the observed shift in methylation level renders DNA susceptible to breaks in this region (Esteller and Herman 2002). Our results also show that the observed changes are limited to the breakpoint region as the assays 1 and 4 located further outside the breakpoint region showed comparable methylation levels in the child, parents, and controls. It must however be noted that the methylation level shown by assays 1 and 2 in the child corresponds only to the wild-type chromosome 1, as this region is deleted on the homologous chromosome.

Study limitations include the small number of sequenced samples in this study and the assessment of somatic cells in the absence of germ cells available for testing.

## Conclusions

Studied patients with monosomy 1p36 exhibited dysregulation of DNA methylation at the 1p36 breakpoint hotspot region. The significantly higher GC content of repetitive elements in this region compared to the rest of the genome suggests the susceptibility of the 1p36 hotspot to DNA methylation changes. Overall, the results point to changes in methylation level as a potential factor contributing to frequent aberrations in the 1p36 region in patients with monosomy 1p36. However, it remains possible that the actual chromosomal rearrangements might have caused changes in DNA methylation level in the patients. Further studies on DNA methylation in monosomy 1p36 will improve the knowledge about the involvement of methylation alterations in the molecular mechanism of chromosomal rearrangements and specifically, monosomy 1p36.

## Supplementary Information

Below is the link to the electronic supplementary material.

Supplementary file1 (PDF 111 KB)

Supplementary file2 (XLSX 314 KB)

Supplementary file3 (XLS 25 KB)

Supplementary file4 (XLSX 12 KB)

Supplementary file5 (XLS 25 KB)

Supplementary file6 (PDF 71 KB)

Supplementary file7 (PDF 67 KB)

## Data Availability

The datasets generated during and/or analyzed during the current study are available from the corresponding author on reasonable request. Final data processing reports created by Bismark are listed in the Mendeley Data repository: 10.17632/8k7r4f36pb.1. The raw data from tBS-Seq are available at 10.17632/5zx4vsjdng.1, 10.17632/jyxxh5vdd5.1, 10.17632/2524cpky8p.1, and 10.17632/ky7ycvbz2k.1.
