# Supplementary material for: DNA methylation dysregulation patterns in the 1p36 region instability
Source: J Appl Genet. 2024 Oct 26;66(3):611–21. doi: 10.1007/s13353-024-00913-9 (PMC12367977; doi:10.1007/s13353-024-00913-9)
Supplement: Supplementary file 1 — Supplementary file1 (PDF 111 KB) [file 13353_2024_913_MOESM1_ESM.pdf]

‘DNA methylation dysregulation patterns in the 1p36 region instability’, *Journal of Applied Genetics*, Swierkowska-Janc J, Kabza M, Rydzanicz M, Giefing M, Ploski R, Shaffer LG, Gajecka M. Correspondence: Prof. Marzena Gajecka, Institute of Human Genetics, Polish Academy of Sciences, Poznan, Poland, gamar@man.poznan.pl

Supplementary Table S1. Monosomy 1p36 patients with deletions and identified breakpoints at the 1p36 rearrangement hotspot (Gajecka et al. 2007). Targeted bisulfite sequencing was performed in samples derived from patients 21, 41, 56, and 62.

Patient number	Sex	Age at diagnosis	Parental origin[a]	Chromosome	Breakpoint localization, nucleotide position from telomere [bp]		Deletion size [Mb]	Comments
					**Start**	**End**		
**Terminal deletions[b]**								
21	F	2 years	maternal	chr1	4 718 457	4 718 658	4.7	Hypomethylated region just upstream the breakpoint
41	F	14 months	paternal	chr1	4 816 367	4 816 575	4.8	
56	F	10 months	maternal	chr1	4 928 346	4 928 635	4.9	Coverage drop at 4,4–4,5 Mb
62	F		maternal	chr1	4 301 903	4 302 140	4.3	
12	M	Birth	paternal	chr1	5 074 742	5 074 892	5.1	
22	F	Unknown	paternal	chr1	4 324 379	4 324 755	4.3	
28	F	1.5 years	maternal	chr1	4 340 446	4 340 672	4.3	
73	M		paternal	chr1	4 510 344	4 510 640	4.5	
07	M	2 years	maternal		4.0	4.5		
08	F	prenatal	paternal		4.5	5.0		
16		1 year	maternal		4.5	5.0		
20	F	unknown	maternal		4.0	4.5		
23	M	2 years	maternal		4.0	4.5		
37	F	1 year	maternal		5.5	6.0		
48	M	8 months	maternal		4.5	5.0		
57	M	2 years	maternal		4.5	5.0		
58	F	19 years	paternal		3.5	4.0		
76	F		maternal		4.0	4.5	4.8	
78	M		unknown (adopted)		4.5	5.0	4.75	

86	F		paternal	5.0	5.5	5.9	
89	F		maternal			4.5	
93	M		paternal			4.5	
123	F	1 year	paternal			5.5	
**Interstitial deletions**[b]							
01	M	11 years	maternal	1.0-1.5	4.5-5.0	3.0 - 4.0	
10	M	birth	maternal	0-0.5	5.5-6.0	5.0 - 6.0	
17	F	1 year	maternal	0-0.5	3.5-4.0	3.0 - 3.5	
138	M		maternal			4.0	
145	M		paternal			4.0 - 6.5	
**Translocations and complex aberrations**[b]							
03	M	unknown	unknown	1.0-1.5,<br>4.0-4.5	3.5-4.0,<br>10.0-10.5		complex - two interstitial deletions
09	F	1 month	maternal	0-0.5,<br>4.0-4.5	3.0-3.5,<br>6.5-7.0		complex - two interstitial deletions
05	M	2 years	maternal	4.5	5.0		derivative +1q
15	F	8 years	paternal	5.0	5.5		derivative +1q
19		prenatal	maternal	5.5	6.0		derivative +15p
55-1	M	1 year	maternal	5.5	6.0		derivative +9q
55-2	F	8 years	maternal	5.5	6.0		derivative +9q
56	F	10 months	maternal	3.5	4.0	4.8	derivative; t(1;17)
63	M	3 years	paternal-hybrid	3.5	4.0		derivative +9q
45	M	birth	maternal	4.0,<br>8.5-9.0	4.5,<br>>10.5		complex - terminal del w/interstitial del
69	F		maternal?	ter-0.5, 1.25 Mb ter trp, 4 Mb dup			complex - del/dup/trp
117	F		maternal	~1.0,<br>3.0	1.5,<br>10		complex - 2 interstitial del
118	F	9 years	maternal?			4.5	der 1 t(1;19)(p36.3;p13.3)
109						~ 5.0	duplication
121	M	10 years	maternal			4.0	duplication

[a]Parental origin of the chromosome with a deletion at 1p36, the deletion is *de novo* in all cases

[b]Breakpoints were localized approximately, confirmed using FISH
