# Supplementary material for: DNA methylation dysregulation patterns in the 1p36 region instability
Source: J Appl Genet. 2024 Oct 26;66(3):611–21. doi: 10.1007/s13353-024-00913-9 (PMC12367977; doi:10.1007/s13353-024-00913-9)
Supplement: Supplementary file 6 — Supplementary file6 (PDF 71 KB) [file 13353_2024_913_MOESM6_ESM.pdf]

‘DNA methylation dysregulation patterns in the 1p36 region instability’, *Journal of Applied Genetics*, Swierkowska-Janc J, Kabza M, Rydzanicz M, Giefing M, Ploski R, Shaffer LG, Gajecka M. Correspondence: Prof. Marzena Gajecka, Institute of Human Genetics, Polish Academy of Sciences, Poznan, Poland, gamar@man.poznan.pl

Supplementary Table S6. Numbers of identified differentially methylated cytosines at the single base and regions at 100 bp window level, comparing individual patients and their parents

Comparison	CpG		DMR_100	
	Hypomethylated	Hypermethylated	Hypomethylated	Hypermethylated
**Child vs Parent 1[a]**				
21C_vs_21M	1115	1162	229	269
41C_vs_41F	846	2147	156	530
56C_vs_56M	259	1811	54	449
62C_3_vs_62M	1710	664	419	136
62C_4_vs_62M	2769	771	752	144
**Child vs Parent 2[b]**				
21C_vs_21F	1367	686	302	128
41C_vs_41M	256	473	53	134
56C_vs_56F	32	318	20	324
**Parent 1 vs Parent 2**				
21M_vs_21F	483	263	124	44
41F_vs_41M	1223	1330	231	241
56M_vs_56F	240	285	118	211

[a]Parent 1 – a parent from whom the chromosome on which the deletion occurred *de novo* was inherited;

[b]Parent 2 – a parent from whom the chromosome without deletion was inherited
