# Supplementary material for: DNA methylation dysregulation patterns in the 1p36 region instability
Source: J Appl Genet. 2024 Oct 26;66(3):611–21. doi: 10.1007/s13353-024-00913-9 (PMC12367977; doi:10.1007/s13353-024-00913-9)
Supplement: Supplementary file 7 — Supplementary file7 (PDF 67 KB) [file 13353_2024_913_MOESM7_ESM.pdf]

‘DNA methylation dysregulation patterns in the 1p36 region instability’, *Journal of Applied Genetics*, Swierkowska-Janc J, Kabza M, Rydzanicz M, Giefing M, Ploski R, Shaffer LG, Gajecka M. Correspondence: Prof. Marzena Gajecka, Institute of Human Genetics, Polish Academy of Sciences, Poznan, Poland, gamar@man.poznan.pl

Supplementary Table S7. Results of pyrosequencing validation of 1p36.32 breakpoint hotspot region – the mean DNA methylation levels obtained in designed assays. Assays 1 and 2 correspond to chromosomal region deleted in this patient in one of the homologous chromosomes 1, while assays 3 and 4 correspond to the unchanged region localized proximally to the breakpoint

**Assay**	**Mean DNA methylation in controls**	**Normal range**	**21C**	**21M**	**21F**
Assay 1	2.01%	0.49 – 3.58%	3.65%	5.37%	19.30%
Assay 2	23.42%	11.94 – 36.34%	15.11%	31.26%	32.40%
Assay 3	37.35%	19.19 – 56.43%	70.82%	31.01%	26.56%
Assay 4	92.94%	90.53 – 95.29%	84.17%	79.92%	87.19%
